# Pulsed Electromagnetic Field Assisted *in vitro* Electroporation: A Pilot Study

**DOI:** 10.1038/srep33537

**Published:** 2016-09-16

**Authors:** Vitalij Novickij, Audrius Grainys, Eglė Lastauskienė, Rūta Kananavičiūtė, Dovilė Pamedytytė, Lilija Kalėdienė, Jurij Novickij, Damijan Miklavčič

**Affiliations:** 1 Vilnius Gediminas Technical University, Institute of High Magnetic Fields, Vilnius, 03227, Lithuania; 2 Vilnius University, Department of Biotechnology and Microbiology, Vilnius, 03101, Lithuania; 3 University of Ljubljana, Faculty of Electrical Engineering, Ljubljana, SI-1000, Slovenia

## Abstract

Electroporation is a phenomenon occurring due to exposure of cells to Pulsed Electric Fields (PEF) which leads to increase of membrane permeability. Electroporation is used in medicine, biotechnology, and food processing. Recently, as an alternative to electroporation by PEF, Pulsed ElectroMagnetic Fields (PEMF) application causing similar biological effects was suggested. Since induced electric field in PEMF however is 2–3 magnitudes lower than in PEF electroporation, the membrane permeabilization mechanism remains hypothetical. We have designed pilot experiments where *Saccharomyces cerevisiae* and *Candida lusitaniae* cells were subjected to single 100–250 μs electrical pulse of 800 V with and without concomitant delivery of magnetic pulse (3, 6 and 9 T). As expected, after the PEF pulses only the number of Propidium Iodide (PI) fluorescent cells has increased, indicative of membrane permeabilization. We further show that single sub-millisecond magnetic field pulse did not cause detectable poration of yeast. Concomitant exposure of cells to pulsed electric (PEF) and magnetic field (PMF) however resulted in the increased number PI fluorescent cells and reduced viability. Our results show increased membrane permeability by PEF when combined with magnetic field pulse, which can explain electroporation at considerably lower electric field strengths induced by PEMF compared to classical electroporation.

Electroporation is most commonly described by creation of transient hydrophilic pores in the cell membrane due to cell exposure to pulsed electric fields (PEF) followed by an increase of membrane permeability for molecules otherwise deprived of transmembrane transport mechanisms^1^,^2^,^3^,^4^. The phenomenon of electroporation allows thus delivery of drugs or other molecules inside the viable cells^5^,^6^,^7^,^8^,^9^. Depending on the treatment intensity, described membrane permeabilization process can be transient and cells survive or can lead to cell death and is then referred to as irreversible electroporation (IRE)^2^,^10^,^11^. Reversible electroporation is used in electrochemotherapy and DNA transfection for DNA vaccination and gene therapy^12^,^13^ while IRE is frequently used as tissue ablation method^14^,^15^ – all these procedures have already made their way into the clinical practice^10^,^11^,^12^,^13^,^14^,^15^,^16^,^17^,^18^. Electroporation is also used in biotechnology^19^, biorefinery^20^ and food processing^21^,^22^.

Independent on the application, the electroporation protocols are constantly being developed to improve the efficacy of the treatment^23^,^24^. The efficient permeabilization can be performed only after careful selection of the pulse parameters, such as the pulse amplitude, shape, pulse duration, repetition rate and the total number of pulses, which have major impact on the outcome of the treatment^25^,^26^,^27^. The parametric analysis of the electrical pulses has been addressed in many studies^28^,^29^,^30^. Pucihar *et al*. has confirmed that the amplitude of the applied electric field that is required to induce the electroporation effects, has an inverse relation with the pulse duration^30^. At the same time the long (ms) high power pulses result in the Joule heating effects and non-desirable electrochemical reactions, which are likely to occur at the electrode–electrolyte interface as a result of the passage of high currents^31^,^32^,^33^. Also taking into account the limitations of the experimental setups, pulse generators and the various electrode configurations, it is sometimes difficult to adjust the parameters to achieve high efficacy of the treatment. The typical protocols for *in vitro* electroporation involve application of the microsecond duration pulse bursts, for example 8 × 100 μs^34^,^35^, which are relatively easy to generate. The derivation of the equivalent pulse parameters is also possible^36^. A tendency for shorter, but high intensity pulse application is observed among the latest scientific reports^15^,^37^,^38^,^39^,^40^,^41^,^42^. Nevertheless, the determination of the optimal parameters is still challenging and application-specific.

Recently, as an alternative method to electroporation by Pulsed Electric Fields, cell membrane permeabilization by Pulsed ElectroMagnetic Field (PEMF) was suggested, which causes biological effects similar to electroporation^43^,^44^,^45^,^46^,^47^. The introduction of the PEMF offers a non-invasive method and an impedance-independent pulse parameters protocols, since the electric field is induced in the target sample by time-varying electromagnetic field, which allows achieving contactless permeabilization^45^. Also in case of the conventional electroporation the voltage breakdown between the electrodes can occur which, however is eliminated completely during electroporation by PEMF.

Presently, the influence of the magnetic field component of the PEMF is poorly understood and electroporation as the permeabilization mechanism is hypothetical, due to the complexity of the treatment methodology and the lack of the experimental works. Currently, it is believed that the pulsed magnetic field induces pulsed electric field inside the sample, which results in the contactless electroporation phenomena. However, the efficacy of the PEMF electroporation is inferior to conventional electroporation^47^ but research in the direction of improving protocols is ongoing^43^,^44^,^45^,^46^,^47^. Due to the high currents up to several tens of kA, which are required for the high magnetic field generation, the Joule heating of the inductors is inevitable^48^,^49^. In our previous work we have shown that the temperature rise during the pulse causes decrease of the cell viability, therefore, the experimental results are hard to interpret, when the Joule heating is not managed^48^.

In 2012 Towhidi *et al*., Shankayi *et al*. have successfully demonstrated that the pulsed electromagnetic fields induced permeabilization of CHO cells^44^,^50^. Also Kardos and Rabussay have used 150–300 μs pulsed electromagnetic fields up to 4 T for intracellular plasmid DNA delivery *in vivo*^45^. However, in all of the above mentioned studies the induced electric field value was at best few V/cm, which is at least two orders of magnitude lower from what is usually reported as the electric field leading to electroporation. In electroporation research in order to cause membrane permeabilization one needs to overcome the threshold transmembrane voltage, which is induced by the electric field to which cells are exposed and this usually is in the range of several hundreds V/cm^51^,^52^.

To address this apparent discrepancy we: 1) designed experiments in which we tested if magnetic pulse by itself can cause membrane electroporation; and 2) tested if we can achieve better electroporation by combined and simultaneous exposure of cells to pulsed electric and magnetic fields, determined by membrane permeabilization assay and cell survival.

## Results

**Figure 1:**
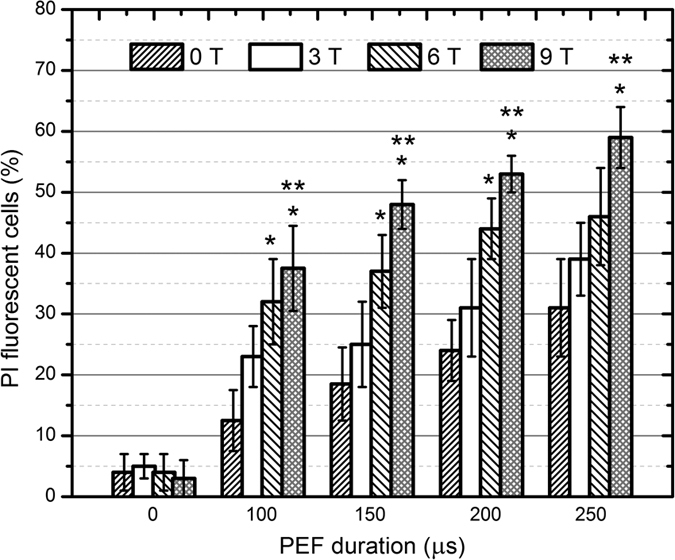
Dependence of the percentage of PI fluorescent *C. lusitaniae* cells on the treatment parameters. The label (*) represents statistically significant difference (*P* < 0.05) between the PEF only treatment and the samples after the PEF + PMF procedures in each instance. The label (**) highlights statistically significant difference (*P* < 0.05) between the PEF + 3 T and the 6, 9 T PEF + PMF instances. The t-test has been used for the statistical significance determination.

The *Saccharomyces cerevisiae* and the *Candida lusitaniae* cells were subjected to a single 100–250 μs electrical pulse of 800 V (8 kV/cm) with and without the concomitant delivery of the magnetic pulse (3, 6 and 9 T). Four independent experimental sessions have been performed. The number of PI fluorescent cells in a sample has been evaluated as the fraction of the fluorescent cells to the total number of cells, expressed as a percentage. The results for *C. lusitaniae* are presented in Fig. 1.

The delivery of the sub-millisecond range magnetic field pulses did not result in any statistically significant changes in the number of the fluorescent cells, independently from the applied magnetic flux density. As it can be seen in Fig. 1 in all of the instances (3, 6, 9 T) the results are within the margins of error and comparable to the control (0 kV/cm, 0 T).

As expected, after the PEF pulse (without the PMF component) the number of the fluorescent PI cells has increased with the duration of the electric pulse, indicating the occurrence of the electroporation phenomenon (up to 31 ± 8% in the 250 μs case). The combined and simultaneous exposure of the cells to pulsed electric and magnetic field resulted in a better electroporation, i.e. the number of PI fluorescent cells increased in each sample (up to 59 ± 5% in the 8 kV/cm 250 μs + 9 T treatment case).

The statistically significant difference (*P* < 0.05) between the PEF only and the samples after the PEF + PMF has been observed in the 6 T and 9 T experimental instances (See Fig. 1, *). On average the 9 T + PEF procedure induced higher number of the cells being permeabilized, compared to the 6 T + PEF case, however the difference was not statistically significant. At the same time the difference between the 3 T + PEF and the 9 T + PEF procedures was significant (*P* < 0.05) in all of the experimental sessions, indicating pulsed magnetic field amplitude influence on the electroporation process (See Fig. 1, **).

**Figure 2:**
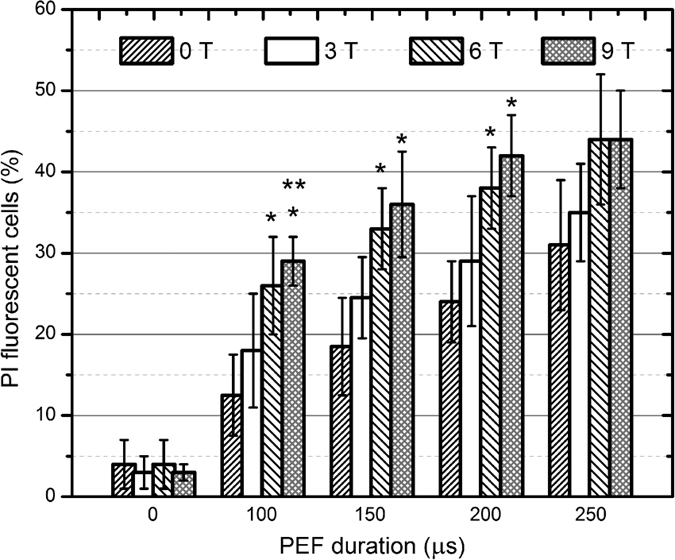
Dependence of the percentage of PI fluorescent *S. cerevisiae* cells on the treatment parameters. The label (*) represents statistically significant difference (*P* < 0.05) between the PEF only treatment and the samples after the PEF + PMF procedures in each instance. The label (**) highlights statistically significant difference (*P* < 0.05) between the PEF + 3 T and the 6, 9 T PEF + PMF instances. The t-test has been used for the statistical significance determination.

Using the same methodology the experiments have been performed with the *S. cerevisiae* α’1 cells. The dependence of the percentage of PI fluorescent *S. cerevisiae* cells on the treatment parameters is summarized in Fig. 2.

In Fig. 2 similar trends and relations for *S. cerevisiae* can be observed. Both yeast cell lines showed increased permeabilization in the 8 kV/cm; 0–6 T treatment range, however in the 9 T, 8 kV/cm experimental instances the number of the PI fluorescent *C. lusitaniae* cells was on average higher than the number of PI fluorescent *S. cerevisiae* cells (*P* < 0.05). We were not able to detect a significant difference between the 9 T + PEF and the 6 T + PEF procedures. Also the difference between the PEF only (8 kV/cm, 250 μs) and the procedure with a concomitant 9 T pulse was not statistically significant too (*P* > 0.05). In the *C. lusitaniae* case a maximum increase by 29% of the PI fluorescent cells was acquired (150 μs, 8 kV/cm + 9 T instance compared to PEF only treatment), however in the *S. cerevisiae* case the maximum improvement by 18% was observed (150–200 μs, 8 kV/cm + 9 T instances).

**Figure 3:**
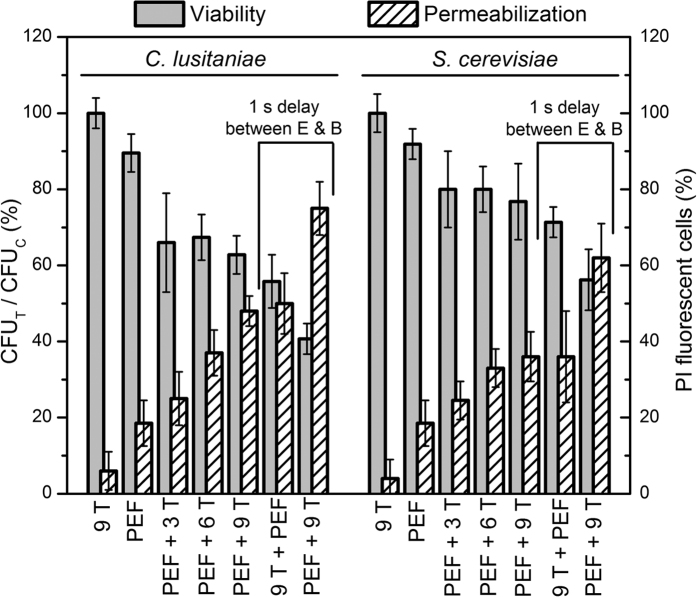
The viability and the permeabilization rate dependence on the treatment parameters, where PEF – single 150 μs, 8 kV/cm square wave pulse.

Further the case when PMF is delivered before the PEF and the opposite case where the PEF treated yeasts are subjected to PMF with a one second delay between the treatment components has been studied. The 150 μs, 8 kV/cm PEF instance was investigated. The viability has been evaluated as a ratio of the number of the colony forming units (CFU) in the sample after treatment (CFU_T_) to the number of the colony forming units in the control sample (CFU_C_) and expressed as a percentage. The results are presented in Fig. 3.

The results are similar for the two cell lines – the PEF only treatment (8 kV/cm, 150 μs) resulted in the average viability decrease of 10 ± 5% compared to the untreated sample, while the PMF only treatment (9 T) showed no statistically significant difference. The combined treatment (PMF + PEF) resulted in a higher viability drop (up to 63 ± 5% in the *C. lusitaniae* case), while the *S. cerevisiae* indicated a weaker response, i. e. lower viability drop (up to 80 ± 6%). The difference in viability between the 3 T, 6 T and 9 T in combined exposure was not statistically significant for both cell lines. The permeabilization data is consistent with the viability results – the number of PI fluorescent cells increased with the decrease of the cell viability.

At the same time the highest number of PI fluorescent cells (lowest viability) has been observed when the PEF treated sample has been subjected to a 9 T magnetic field after a 1 s delay. In the opposite case when the PMF is delivered before the PEF there was no statistically significant difference compared to the concomitant delivery of PEF + 9 T. Both cells lines showed a similar response tendency (viability and permeabilization rates), while the *C. lusitaniae* was on average more susceptible to the combined treatment.

We have also evaluated the fluorescence levels of individual cells in the digitized images using Lucia cytogenetics imaging software (Laboratory Imaging, Czech Republic). However, the difference in the mean fluorescence values of cells between the PEF only, simultaneous PEF + 9 T or delayed PEF + 9 T exposures were not statistically significant (P > 0.05). The generality of the observation has been assessed based on 6–10 images for each separate experimental instance (50 + individual cells).

**Figure 4:**
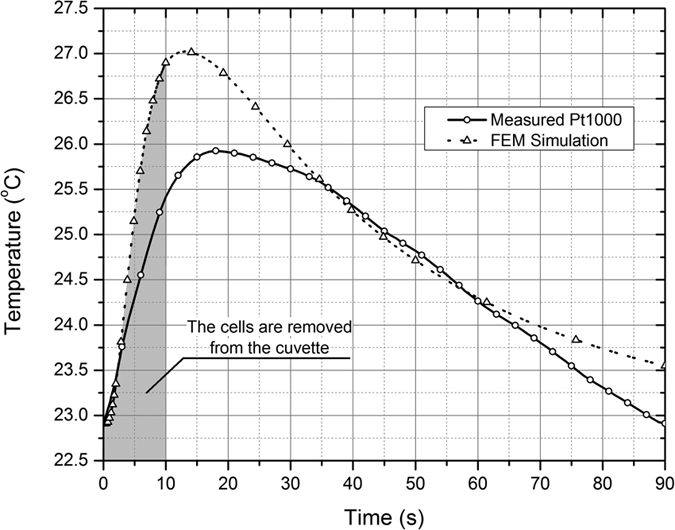
Temperature rise in the cuvette after the 9 T + 8 kV/cm, 250 μs pulse.

We also investigated Joule heating by determining the temperature rise in the electroporation cuvette since heating reported to be an important factor during PEMF when the kA range currents are applied^45^,^48^,^49^. The temperature rise was evaluated both by numerical calculations (FEM simulations) and experimentally with a filled cuvette using the Pt1000 platinum temperature sensor. The results are presented in Fig. 4.

As it can be seen in Fig. 4 the temperature rise due to the Joule heating and the induced currents did not exceed 3 ± 1 °C. The experimental and the results of numerical calculations are in good agreement. In addition, cells have been removed from the cuvette within 10 s (highlighted in the graph) after the pulse for staining with PI, which further minimized any possible thermal influence.

## Discussion

In this paper, we have designed experiments in which we tested if sub-millisecond magnetic pulse by itself can cause membrane electroporation in yeast and if we can achieve better electroporation by combined and simultaneous exposure of cells to pulsed electric and magnetic fields as determined by membrane permeability assay and cell survival. We showed that the single sub-millisecond magnetic field pulses up to 9 T do not cause membrane permeabilization of yeast cells or the effect is beyond the detection ranges of the PI fluorescence microscopy. We believe that the result was due to low *dB*/*dt*, thus low induced electric field value during the single magnetic field pulse. The results are in agreement with the data by *Anton-Leberre et al.*, where the high magnetic field pulses, but by several orders of magnitude lower *dB*/*dt* were used^53^. Therefore, we concluded that the induced electric field and consequently induced transmembrane voltage during the sub-millisecond magnetic field pulses up to 9 T is below the threshold for membrane electroporation in the yeast cells.

The conventional PEF resulted in the uptake of PI by the yeast cells, which as expected increases with the increased pulse duration. However, the effect of the simultaneous exposure of cells to pulsed electric and magnetic fields has been of particular interest. We demonstrate that the number of PI fluorescent cells increased in each sample exposed to the combined PEF + PMF. The hypothetical mechanism of the observed increase could be the interaction of the charged/polarized particles with the high magnetic field that is present in the cuvette, resulting in additional motion of the ions in the medium. Also even though the *Saccharomyces* and *Candida* have very similar responses to many environmental stresses, including that of starvation^54^, in our work the number of *C. lusitaniae* PI fluorescent cells was on average higher than the number of permeabilized *S. cerevisiae* cells. The results were consistent with the viability data – the growth inhibition of the *C. lusitaniae* was on average higher compared to the *S. cerevisiae* in all PMF + PEF combinations. We assume that the differences in the response may be influenced by the differences in the cell-wall structure between the yeast, where such parameters as thickness, porosity and the macromolecular composition vary considerably^55^. The reports in which *Candida* proves as more sensitive specie to the combinatorial stress compared to *S. cerevisiae* can also be found^56^.

The viability results, as a response to treatment using different parameters was consistent for both cells lines. We have observed a statistically significant decrease of viability during simultaneous exposure of cells to PEF + PMF compared to the PEF only exposure, however the viability rate did not depend on the magnetic field value indicative of a saturated effect for both yeasts. At the same time the number of permeabilized cells (PI fluorescent) showed an incremental tendency with increase of the PMF amplitude. The result could indicate an increased number of reversibly permeabilized cells, which depends on the PMF amplitude. However, such an assumption is not necessarily true since the mechanism of effect is still hypothetical. It is also possible that the PEF + PMF exposure triggers both necrotic and apoptotic pathways following by an early necrotic and delayed apoptotic death (several hours), which at the end results in a similar long-term survival. Such a phenomenon has been already reported in the conventional nanosecond electroporation^57^. In our case we have performed the pilot study using the conventional PI dye to show the proof of concept and presence of interaction between PEF and PMF, while the mechanism of cell death remains the topic of future works.

It was also shown that the delayed (1 s) concomitant delivery of PEF and PMF pulses, when the PEF pulse is followed by a 9 T PMF results in a higher number of permeabilized cells and a higher viability drop (P < 0.05) compared to the simultaneous PEF + PMF delivery. The results indicate an effect similar to electrosensitization, when the splitting of the train of electric field pulses by a delay enhances the effect severalfold^58^. As a result the lethality of treatment could be increased simply by control of the delay between the pulses while the total energy of the exposure is identical^58^,^59^. The protocol when the PMF component is delivered before PEF showed no statistically significant difference compared to the simultaneous PEF + PMF procedure. Based on the results obtained and presented we can conclude that the induced electric field value can not be considered as a sole factor influencing permeabilization during PEMF treatment as it was assumed previously^44^,^45^,^46^,^47^. Further investigation of the permeabilization phenomenon obtained by the pulsed electromagnetic field is required to provide a better understanding of the interaction. Nevertheless, this observed interaction of pulsed magnetic and electric field can at least offer partial explanation of PEMF permeabilization, which is observed at considerably lower electric field strength (in the range of V/cm) as compared to classical electroporation where the 2–3 orders of magnitude higher electric fields are reported to cause membrane electroporation.

## Methods

### Electroporation setup

The 1 kV, 50 A, 5 μs – 5 ms square wave electroporator that was developed in the High Magnetic Field Institute of Vilnius Gediminas Technical University (Vilnius, Lithuania) was used in this study^60^. The 960 μF capacitor array was used in the prototype for the energy accumulation to ensure the square wave pulse waveform.

As a load a standard commercial electroporation cuvette with 1 mm gap between the aluminum electrodes was used (BTX, Cuvette plus, Nr. 610, San Diego, USA).

### Magnetic field setup

For the pulsed magnetic field generation the 40 kA, 4 kV pulse generator based on the high power thyristor (silicon controlled rectifier) switch and the crowbar circuit has been used^61^. As a load the solenoid type inductor, that was developed specifically for the task was connected. The inductor has been wounded using 1.5 mm diameter enameled copper wire with the additional epoxy encapsulation. The external 1 cm thick steel shield has been introduced to enforce the structure and prevent any deformation due to the Lorentz forces during the high power magnetic field pulse. The inner diameter of the inductor was 1.7 cm to match the BTX cuvette. All the other parameters of the solenoid are summarized in Table 1.

**Figure 5:**
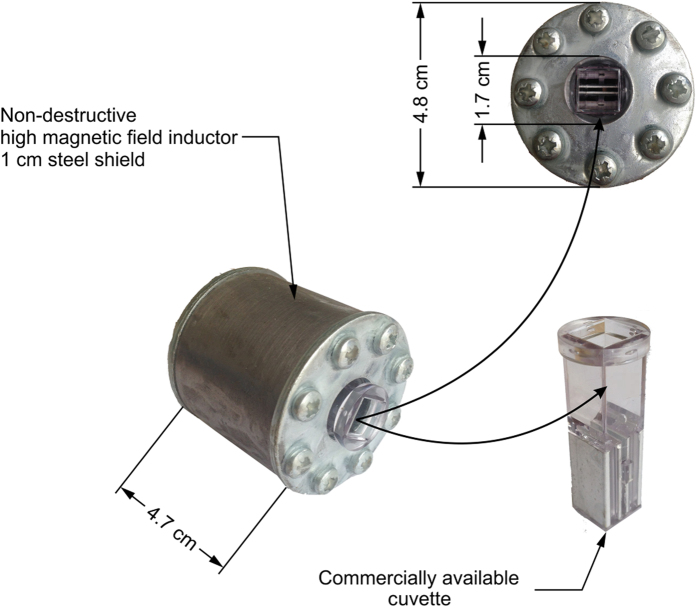
Photograph of the inductor with integrated electroporation cuvette.

The photograph of the developed inductor with the integrated commercial electroporation cuvette is shown in Fig. 5.

**Figure 6:**
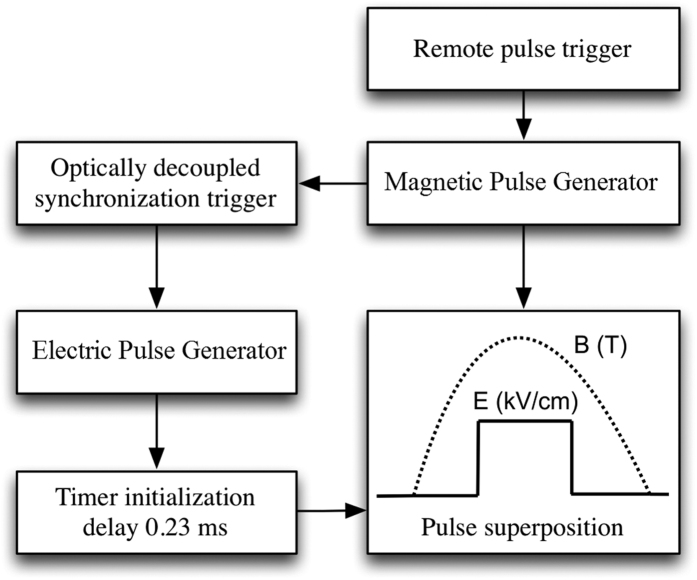
Block diagram of the experimental setup.

The cuvette has been positioned inside the inductor for the cells to be exactly in the center of the coil. In order to subject the biological cells to the pulsed magnetic and electric fields simultaneously, the synchronization of the magnetic and electric field generators was enabled by the setup depicted in the block diagram, which is shown in Fig. 6.

The user using the remote control triggers the magnetic field setup, which starts to discharge the 5.4 mF capacitor array through the developed inductor. At the same time the optically decoupled 5 V synchronization trigger initializes the electroporator unit. The electrical pulse through the cuvette was generated when the magnetic field value is at maximum (9 T ±5%), i.e. 0.23 ms delay between the magnetic and the electric field pulses was introduced. The XMEGA128A3U (Atmel, USA) 8-bit microcontroller was used for the delay implementation. As a result the up to 250 μs square wave electrical pulse can be generated when the magnetic field value in the cuvette is 3, 6 or 9 T ±5%.

**Figure 7:**
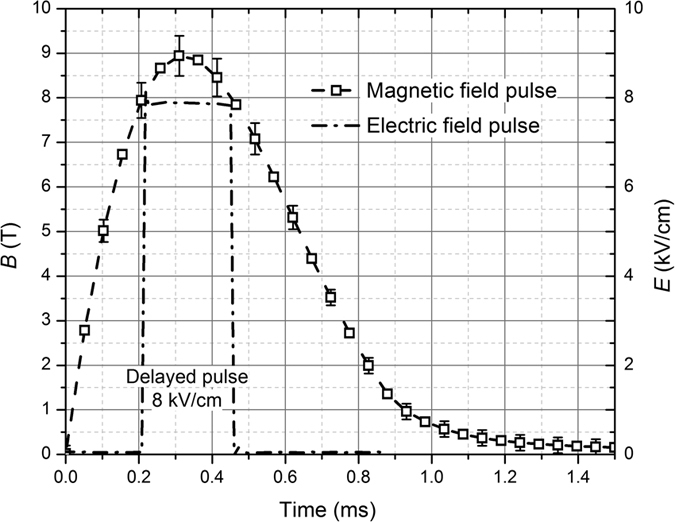
Experimental electric field and magnetic field pulses.

The measured magnetic and electric field pulses are shown in Fig. 7. The pulses have been measured using a calibrated B-dot sensor, which was positioned in the center of the coil for the axial magnetic field measurement^62^ (VGTU, Lithuania). For pulse acquisition the DPO4034 digital oscilloscope (Tektronix, Oregon, USA) was used, the data was post-processed using OriginPro software (OriginLab, Northhampton, MA, USA). In order to achieve the 9 T magnetic flux density (*B*), the capacitors (5.4 mF) of the generator had to be charged to 960 V, resulting in a 2.5 kJ pulse.

The 8 kV/cm electric field strength (*E*) has been achieved using the 800 V electrical pulse in a 1 mm electrode gap cuvette. The spatial distribution of the magnetic field was evaluated using the finite element method (FEM) analysis in Comsol Multiphysics software (COMSOL, Stockholm, Sweden)^63^,^64^. The coil was driven by single current pulse described as a time function with the peak amplitude of 10.2 kA and duration of 1.2 ms (Fig. 7). The triangular mesh consisted of 35764 finite elements. The maximum edge size of each finite element varied from 0.001 to 0.5 mm with the maximum growth rate of 1.679.

**Figure 8:**
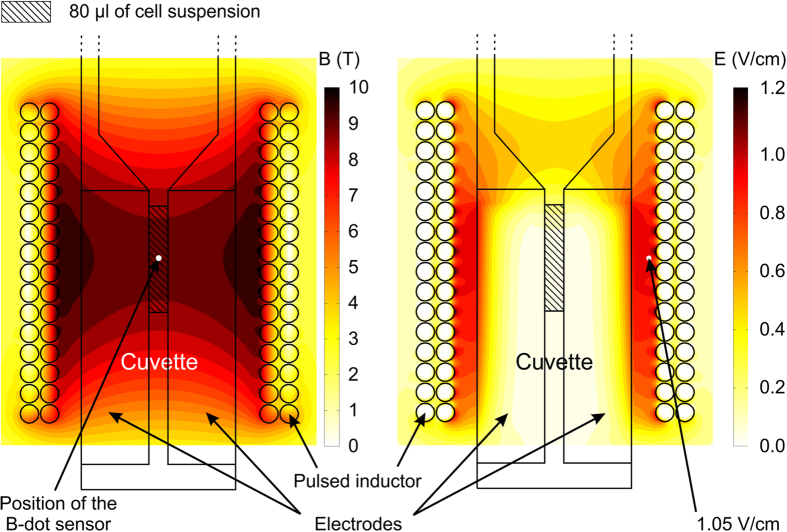
Spatial distribution of the maximum magnetic and the induced electric fields in the coil.

The spatial distribution of the magnetic and the induced electric field (maximum dB/dt) in the coil is shown in Fig. 8. The point where the B-dot sensor has been positioned for the experimental measurement of the magnetic field is highlighted in the figure. Based on the spatial distribution of the magnetic field it has been confirmed that the proposed applicator structure (inductor + cuvette) allowed achieving no less than 98% magnetic field homogeneity in the cell medium (80 μl cuvette volume). The peak induced electric field value during each magnetic field pulse was in the range of 0.35, 0.7 and 1.05 V/cm for the 3, 6, 9 T pulses, respectively.

However, since the cells were positioned in the coil center (See Fig. 8) the induced electric field in the medium was only a fraction of the peak value, i.e. less than 0.03 V/cm. Therefore, it has been assumed that the influence of the induced electric field on the cells was negligible compared to the simultaneously applied 8 kV/cm electric field pulse.

### Temperature measurement

The temperature measurement has been performed under experimental conditions using Pt1000 sensor, which was positioned in the cuvette (submerged into 1 M sorbitol suspension). The data from the sensor has been processed using a computerized setup (GPIB interface) based on Tektronix DMM4050 multimeter (Tektronix, Oregon, USA). The 9 T magnetic field and maximum duration 250 μs 0.8 kV electrical pulses have been generated, which corresponds to the highest intensity treatment when the maximum temperature rise is expected. The steel shield of the inductor served both as protection from the deformation and as a heatsink, resulting in thermal inertia and thus slowing temperature rise in the cuvette (See Fig. 4).

### Biological Cells

The *Saccharomyces cerevisiae* α’1 and *Candida lusitaniae* C18 strains were used in experiments. *Saccharomyces cerevisiae* α’1 was obtained from the collection of microorganisms of Nature Research Centre (Lithuania). *C. lusitaniae* was isolated from the skin of the patient with clinical diagnosis of atopic dermatitis and identified applying assimilation test api ID32C (bioMerieux sa, France)^65^. Informed written consent was obtained from all subjects. Physiological identification was confirmed by 26S rDNA sequences analysis^66^. The methods were approved by the Lithuanian Bioethics Committee, 2007 07 19, license number 24. All applicable international, national, and/or institutional ethical guidelines were followed. For the experiments *S. cerevisiae* and *C. lusitaniae* cells were grown on the rich YPD medium (2% glucose, 2% peptone, 1% yeast extract and 1% agar). After 48 hours growth at 30 °C the yeast cells were harvested and 3 times washed with 1 M sorbitol. For further experiments the 10^10^ cells/ml density suspension was prepared in 1 M sorbitol. The 80 μl samples of the suspension were used for experiments. After the treatment the yeast cells were removed from the cuvette and within 10 s stained with 50 μM propidium iodide (PI). After 5 minutes staining at room temperature propidium iodide was removed by centrifugation (5 min, 2000 rpm) and the cells were 3 times washed with PBS buffer. The cells were applied on the polylysine coated slides and analyzed using the light and fluorescent microscopy at wavelength of 550 nm. For the fluorescent microscopy Eclipse 80i microscope (Nikon, Japan) was used. Images were captured by CCD KOOL1300Q camera (VDF Voskühler, Germany) and processed using Lucia cytogenetics imaging software (Laboratory Imaging, Czech Republic). Fluorescent stained cells were counted as PI positive (permeabilized) and unstained cell as PI negative (non-permeabilized).

## Additional Information

**How to cite this article**: Novickij, V. *et al*. Pulsed Electromagnetic Field Assisted *in vitro* Electroporation: A Pilot Study. *Sci. Rep.* **6**, 33537; doi: 10.1038/srep33537 (2016).

## Figures and Tables

**Table 1:** The parameters of the pulsed inductor.

Parameter	Value
inner coil diameter	1.7 cm
outer coil diameter	2.6 cm
shield inner diameter	2.8 cm
shield outer diameter	4.8 cm
coil inductance	13.7 μH
coil active resistance	0.035 Ω
number of windings per layer	16
number of layers	2
